# First Insights into the Bacterial Diversity of Mount Etna Volcanic Caves

**DOI:** 10.1007/s00248-023-02181-2

**Published:** 2023-02-08

**Authors:** Giuseppe Nicolosi, José L. Gonzalez-Pimentel, Elena Piano, Marco Isaia, Ana Z. Miller

**Affiliations:** 1 Department of Life Sciences and Systems Biology, University of Turin, Turin, Italy; 2 Centro Speleologico Etneo, Catania, Italy; 3 HERCULES Laboratory, University of Évora, Évora, Portugal; 4 Instituto de Recursos Naturales Y Agrobiologia de Sevilla (IRNAS-CSIC), Seville, Spain

**Keywords:** Bacteria, Microbial mats, *Actinomycetota*, Lava tubes, 16S rRNA gene analysis

## Abstract

While microbial communities in limestone caves across the world are relatively understood, knowledge of the microbial composition in lava tubes is lagging behind. These caves are found in volcanic regions worldwide and are typically lined with multicolored microbial mats on their walls and ceilings. The Mount Etna (Sicily, S-Italy) represents one of the most active volcanos in the world. Due to its outstanding biodiversity and geological features, it was declared Natural Heritage of Humanity by the UNESCO in 2013. Despite the presence of more than 200 basaltic lava tubes, the microbial diversity of these hypogean systems has never been investigated so far. Here, we investigated bacterial communities in four lava tubes of Mount Etna volcano. Field emission scanning electron microscopy (FESEM) was carried out for the morphological characterization and detection of microbial features. We documented an abundant presence of microbial cells with different morphotypes including rod-shaped, filamentous, and coccoidal cells with surface appendages, resembling actinobacteria reported in other lava tubes across the world. Based on 16S rRNA gene analysis, the colored microbial mats collected were mostly composed of bacteria belonging to the phyla *Actinomycetota*, *Pseudomonadota*, *Acidobacteriota*, *Chloroflexota*, and *Cyanobacteria*. At the genus level, the analysis revealed a dominance of the genus *Crossiella*, which is actively involved in biomineralization processes, followed by *Pseudomonas*, *Bacillus*, *Chujaibacter*, and *Sphingomonas*. The presence of these taxa is associated with the carbon, nitrogen, and ammonia cycles, and some are possibly related to the anthropic disturbance of these caves. This study provides the first insight into the microbial diversity of the Etna volcano lava tubes, and expands on previous research on microbiology of volcanic caves across the world.

**Supplementary Information:**

The online version contains supplementary material available at 10.1007/s00248-023-02181-2.

## Introduction

In the last 20 years, considerable effort has been made to shed light on the microbial communities of karst caves, particularly speleothems and walls hosting wall paintings [1, 2]. In contrast, the microbiology of lava tubes has received much less attention.

Microbial communities in lava tubes grow forming extensive colored biofilms on speleothems and walls [3], similarly to what happens in karst caves [4, 5]. Recent studies conducted in lava tubes revealed a highly diverse microbiome, dominated by new microbial life forms and interactions differing from those occurring on the surface [3, 6]. They can thrive in these harsh oligotrophic environments by interacting with minerals and inducing biomineralization processes [7].

When considering the composition of such colored microbial mats, evidence available in the literature showed that these colonies are mainly composed of metabolically active *Actinomycetota*, as revealed by cDNA analysis of yellow colonies from a lava tube in La Palma, Canary Island, Spain [8]. In general, *Actinomycetota* and *Proteobacteria* are the two most abundant groups of microorganisms in lava tubes (e.g., Northup et al. [9]; Hathaway et al. [10]). One of the most complete studies to date on *Actinomycetota* was carried out by Riquelme et al. [3], in volcanic caves from the USA, Canada, and Portugal, highlighting the importance of caves as a source of new species of *Actinomycetota* [3, 11].

While we have some understanding on geomicrobiology of lava tubes from Hawaii, New Mexico and California, USA [6, 9, 10], Azores, Portugal [12], Easter Island, Chile [7, 13], British Columbia, Canada [3], Galapagos Islands, Ecuador [14–16], and La Palma Island, Spain [17, 18], no data are available on the microbial communities growing in lava tubes of Mount Etna (Sicily, S-Italy). Mount Etna represents one of the most active volcanos in the world [19], proclaimed a Natural Heritage Site by the UNESCO since 2013 due to its outstanding biodiversity and unique geological features. Its volcanic activity dates back more than 500,000 years ago. From about 57,000 years ago, the intense eruptive activity formed the 3600-m-high Ellittico stratovolcano, and from about 15,000 years ago, the Mongibello volcano, whose 357 lava flows cover 88% of the entire surface of Mount Etna [20].

Over 200 basaltic lava tubes are present around the Mount Etna volcano [21]. They generally form when the outer surface of lava channels cools more rapidly forming a hardened crust, while the inside continues to flow until it finishes draining and a tunnel is generated. The viscosity of the lava flow, which is related to the chemical composition of the molten rock, dissolved gases, temperature, and flow velocity, hence controls the formation of lava tubes [22]. The surface of “aa” lava flows is fragmental, spinose, and generally clinker, whereas “pahoehoe” lava flows are smooth. The formation of either is strongly influenced by the viscosity of the lava during eruptions [23]. Although it is believed that lava tubes only form on pahoehoe lavas, the formation of caves on Etna frequently occurs in “*aa*” lava, and some of the tubes that are considered to have formed on pahoehoe formed in large “*aa*” lava flows [24]. The composition of these caves corresponds mainly to basaltic lava, formed by silicates, such as clinopyroxene, plagioclase, and olivine, in addition to iron minerals, intercalated with carbonates and opal of biogenic origin [7, 13, 22].

Despite the great scientific interest determined by the frequent eruptive events occurring on the volcano, both on the summit and lateral flanks, and the peculiarity of its flora [25] and fauna (e.g., Caruso [26]; Magrini et al. [27]; Ebejer and Nicolosi [28]), research focusing on microorganisms is still lagging behind (but see Hopkins et al. [29]; Badalamenti et al. [30]), and study focused on microorganisms living on lava tubes has never been performed so far.

Here we aimed at providing the first microbiological assessment of biofilms coating the walls of four lava tubes located in Mount Etna Park (Sicily, Italy). The characterization of microbial communities from Etna lava tubes is fundamental to the understanding of this uncharted microbial diversity, and also contributes to the preservation of these unique geoheritage sites, recently receiving major interest from society both for their scientific and touristic value [31]. Therefore, reasonable protective and scientific measures should be applied to improve their value [32].

## Materials and Methods

### Studied Site and Sample Collection

**Fig. 1:**
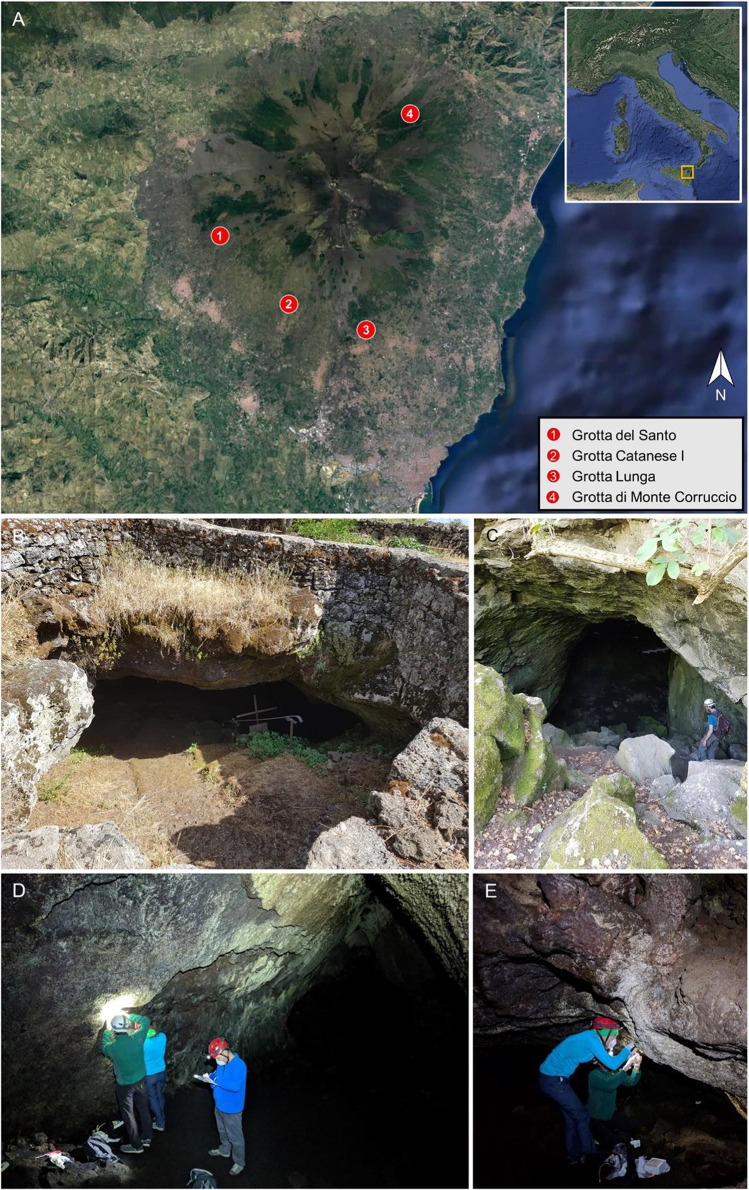
Map of Mount Etna (Sicily, Italy) with the location of the sampled lava tubes (**A**). Source: Google Maps [33]. **B** Entrance of “Grotta del Santo.” **C** Entrance of “Grotta Catanese I.” **D** General view of “Grotta Lunga.” **E** General view of “Grotta di Monte Corruccio”

The Etna volcano is located 20 km north of the city of Catania (15° 0′ E, 37° 43.8′ N), in Sicily, Italy (Fig. 1).

In June 2018, we performed a sampling campaign in four lava tubes of the Etna volcano, namely “Grotta del Santo,” “Grotta Catanese I,” “Grotta Lunga,” and “Grotta di Monte Corruccio.” The main features of each cave are reported in Table S1. Replicate samples of colored microbial mats were aseptically collected using sterile scalpels and stored in sterile 1.5-ml microtubes. Each microbial mat sample was collected from an area of approximately 20 cm^2^. All samples were stored at 4 °C until transportation to the lab. Samples for DNA-based analysis were stored at − 80 °C until laboratory procedures were performed. Samples for microscopy observations were immediately processed upon arrival to the lab.

“Grotta del Santo” or “Grotta di San Nicola” (registered with reference SICT1032 at the “Catasto delle Grotte della Sicilia” of the “Federazione Speleologica Regionale Siciliana”) is a lava tube divided into several galleries reaching a total length of over 900 m. Lava flow here is attributed to a time interval of 15,000–3930 ± 60 years [20]. Results of bulk rock analyses for major (wt%) and trace (ppm) elements derived from Lanzafame and Ferlito [34] are presented in Table S1. The cave entrance has an altar erected in memory of Saint Nicola Politi, patron saint of Adrano (CT), who, according to tradition, lived in this place from 1134 to 1137. The cave is frequently visited, being a destination for religious pilgrimages (Fig. 1B). Although the presence of an iron gate, the cave is easily accessible. Within the lava cave galleries, four sampling sites comprising extensive yellow, white, and beige microbial mats coating the cave walls (designated GS_1A, GS_2A, GS_2B and GS_4; Table S2) were collected and analyzed.

“Grotta Catanese I” (SICT1037) is a lava cave that originated during the “Monte Arso” eruption, about 500 B.C. It is characterized by a large entrance hall, from which a narrow tunnel with about 70-m-long branches off laterally (Fig. 1C). The cave is freely accessible and occasionally frequented by local visitors. Grey and reddish colonies (designated GC1_1A and GC1_2; Table S2) were collected in the twilight zone.

“Grotta Lunga” or “Grotta di Monpeloso” (SICT1029) is a 55-m-long outflow tunnel that originated from the eruptive apparatus of “Monpeloso” formed in 300 ± 100 AD (Fig. 1D) [20]. Results of bulk rock analyses for major (wt%) and trace (ppm) elements derived from Matteoni [35] are presented in Table S1. Here we observed white and grey colonies (designated GL_1 and GL_3) along the main gallery (Table S2). The cave is freely accessible and frequently visited by tourists.

“Grotta di Monte Corruccio” (SICT1056) is an outflow tunnel partially contained in the effusive eruptive apparatus of the homonymous mount formed in a time interval of 15,000–3930 ± 60 years [20]. It has a total length of about 80 m (Fig. 1E). The cave is freely accessible and occasionally frequented by tourists. Here we observed yellow, white, and beige colonies (designated GMC_1, GMC_2, GMC_3, and GMC_4) along the cave galleries (Table S2).

Despite no information is available on the precise number of tourists entering each cave, based on an estimate of the tourist frequentation and the presence of waste due to visits, we can sort the four caves in two categories: those that experience low tourist use (“Grotta Catanese I” and “Grotta di Monte Corruccio”) and the ones experiencing higher tourist use (“Grotta del Santo” and “Grotta Lunga”).

### Morphological Characterization by Microscopy Techniques

Small fragments of each sample collected in the four lava tubes were observed using a Zeiss Discovery V8 stereomicroscope (200 × maximum magnification) coupled to a MOTICAM 10.0 system to perform a detailed macroscopic examination of the sample surface.

Subsequently, samples were examined by field emission scanning electron microscopy (FESEM) using a high-resolution FEI Teneo SEM (FEI Company, Eindhoven, The Netherlands) equipped with an Oxford X-ray energy dispersive spectroscopy (EDS) detector for characterizing the microtopography of the samples and detect microbial cells.

Air-dried bulk samples with microbial mats were directly mounted on a sample stub and sputter coated with a thin platinum film, with an acceleration voltage of 5 kV, using the SE detector.

### Statistical Analysis

The statistical analyses were performed in R [36]. The taxonomic richness measured at the order level for the four caves was tested by means of a generalized linear model (GLM) with a Poisson error distribution [37]. Differences among caves were then tested with the Tukey’s post hoc test, with the function “glht” from the multcomp package [38].

### Taxonomic Characterization of Microbial Communities

Molecular biology techniques based on 16S rRNA gene analysis were conducted for the identification of the bacterial communities present in the Etna lava tube samples.

Genomic DNA was extracted from 12 samples collected in the Etna lava tubes using the DNeasy PowerLyzer PowerSoil Kit according to the manufacturer’s protocol (Qiagen), and quantified using a Qubit 4.0 fluorometer (Invitrogen). Sequencing libraries of the V3-V4 hypervariable region of the 16S rRNA gene were prepared according to the Illumina 16S Metagenomic Sequencing Library protocols. The gDNA input (2 ng) was amplified by PCR using the universal primer pair 314F (5′- Illumina overhang- CCTACGGGNGGCWGCAG -3′) and 805R (5′- Illumina overhang- GACTACHVGGGTATCTAATCC -3′) with Illumina adapter overhang sequences. The thermocycling conditions were as follows: 3 min at 95 °C, 30 s at 55 °C, and 30 s at 72 °C, followed by a 5-min final extension at 72 °C. The PCR products were then purified with AMPure beads (Agencourt Bioscience, Beverly, MA). Two microliters of each purified product was PCR amplified for final library construction containing the index using NexteraXT Indexed Primer, under the same thermocycling conditions as mentioned before, except for 10 cycles. After purification with AMPure beads, the final purified products were quantified using qPCR according to the qPCR Quantification Protocol Guide (KAPA Library Quantificatoin kits for Illumina Sequecing platforms) and qualified using the TapeStation D1000 ScreenTape (Agilent Technologies, Waldbronn, Germany). The purified amplicons were then sequenced using the MiSeq™ platform (Illumina, San Diego, USA) by Macrogen Sequencing Services (Korea).

The raw data obtained from Illumina platform MiSeq, for producing 300 PE reads, was initially quality checked, trimmed, and clustered in ASVs using QIIME2 [39] with DADA2 [40]. ASV is a higher-resolution analogue of the traditional OTU table, which records the number of times each exact amplicon sequence variant was observed in each sample. Taxonomic identification was carried out using SILVA database v.132. Alpha diversity metrics (Shannon’s index and Pielou’s evenness) of bacterial communities were also calculated to investigate community heterogeneity within sample diversity. The raw reads wear deposited into the NCBI Sequence Read Archive (SRA) database under the project id PRJNA914266.

## Results and Discussion

### Microscopy Observations

Under the stereomicroscope, samples showed colored stains coating the rock substrate or associated with mineral grains (Table S2). There were notable differences in colony morphology, texture, and size.

**Fig. 2:**
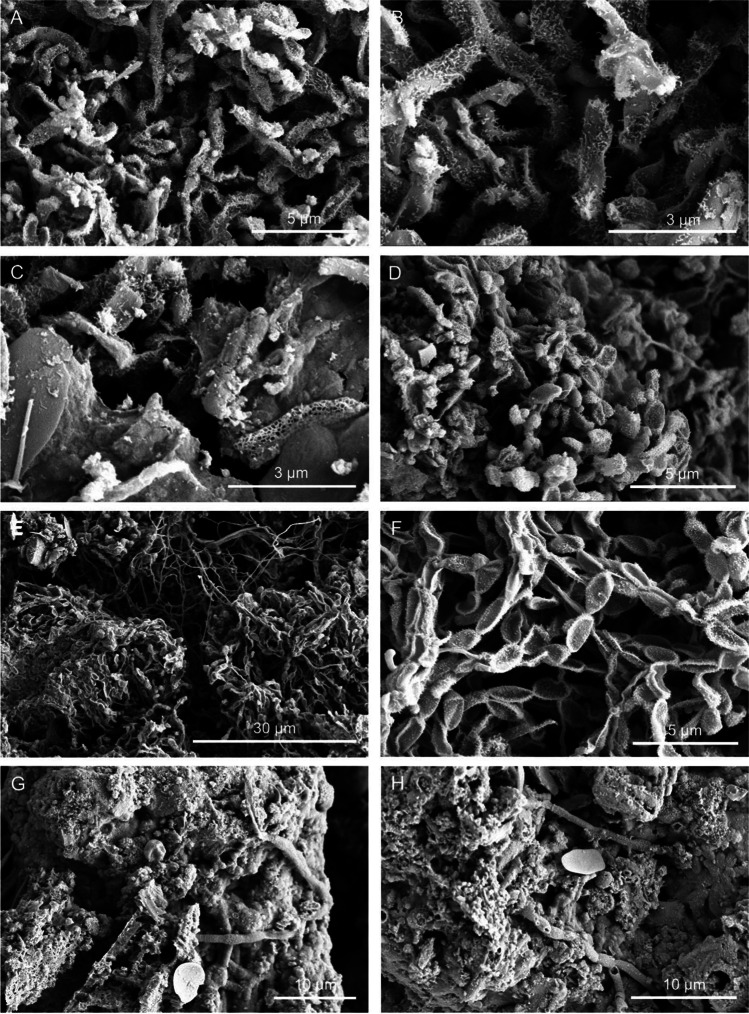
Field emission scanning electron microscopy images of “Grotta del Santo” (GS) samples. Representative FESEM images of the studied samples, depicting **A** biofilm of filamentous actinobacteria-like cells (GS_1A); **B** close-up view of the spore chains with cell surface appendages (GS_1A); **C** fragments of reticulated filaments sparsely distributed within the sample (GS_1A); **D** biofilm of actinobacterial cells with spiny ornamentation (GS_2A); **E** dense network of actinobacteria cells with spiny ornamentations intermingled with Actinobacteria-like hyphae, and **F** spore chains with spiny protuberances on their surface (GS_2B); and **G**, **H** filamentous microbial structures with smooth surfaces in close association with the mineral substrate (GS_4)

**Fig. 3:**
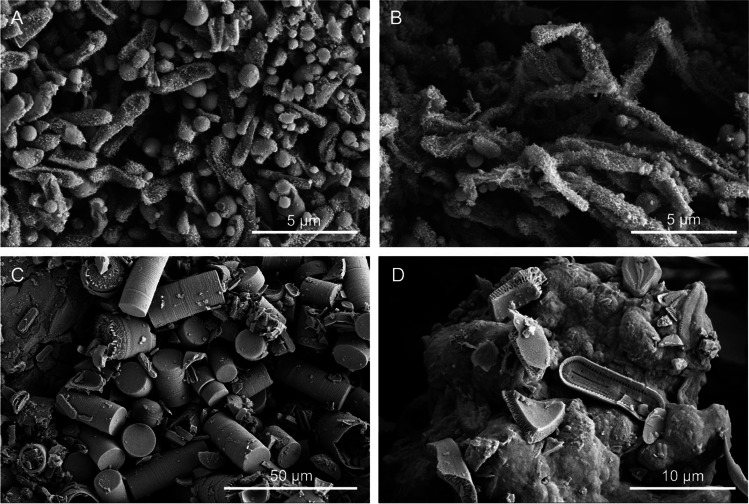
Field emission scanning electron microscopy images of “Grotta Catanese I” (GC) samples. Representative FESEM images of the studied samples, depicting **A** Actinobacteria-like coccoid (with 1-μm diameter) and rod-shaped cells with spiny ornamentation (GC1_1A); **B** filaments with hairy ornamentation, intermingled with coccoid cells (GC1_1A); and **C**, **D** clusters of the diatom *Orthoseira roeseana* and *Humidophila* (GC1_2)

**Fig. 4:**
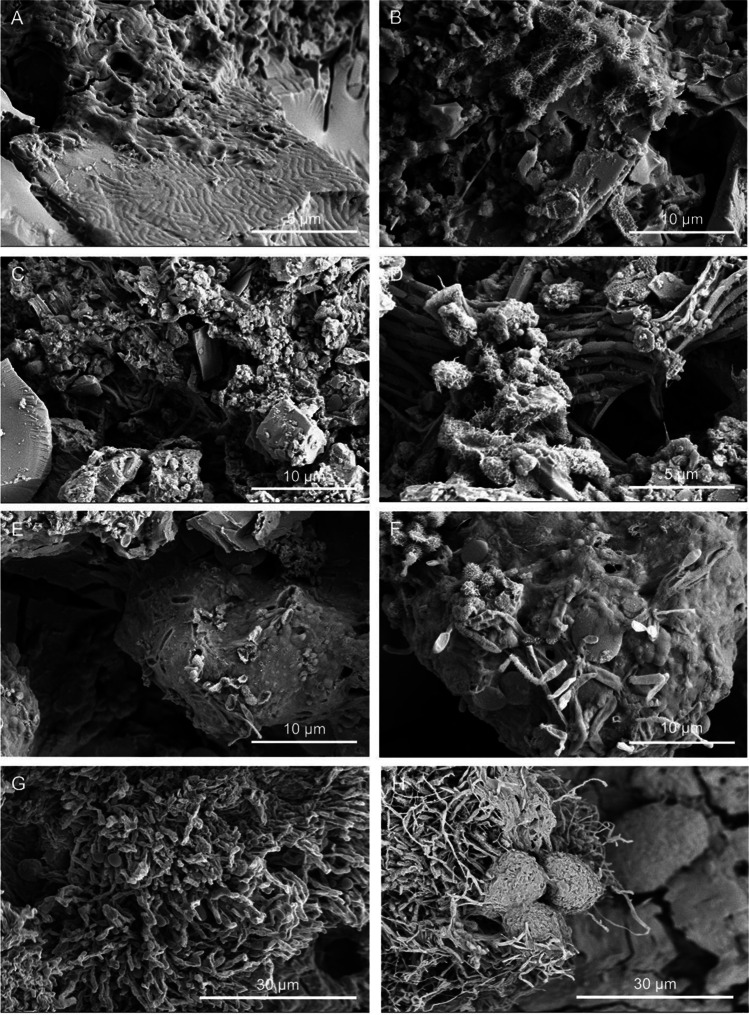
Field emission scanning electron microscopy images of “Grotta di Monte Corruccio” (GMC) samples. Representative FESEM images of the studied samples, depicting **A** rod-shaped cells embedded in the EPS layer (GMC_1); **B** actinobacteria-like spores with hairy filamentous forms (GMC_1); **C** filamentous cells associated with mineral grains (GMC_2); **D** Actinobacteria-like cells with appendages (GMC_2); **E** microbial imprints showing cell-like structures (GMC_4); **F** clusters of Actinobacteria-like cells with spiny ornamentation (left) and rod-shaped bacteria with spiny ornamentation impregnated in EPS (right) (GMC_4); **G** dense masses of bacterial cells, mainly rod-shaped mats showing filaments covered with pili/fimbrae with spheroid shapes emerging from the ends (GMC_5); and **H** Ca-rich spheroids closely associated with filamentous cells (GMC_5)

**Fig. 5:**
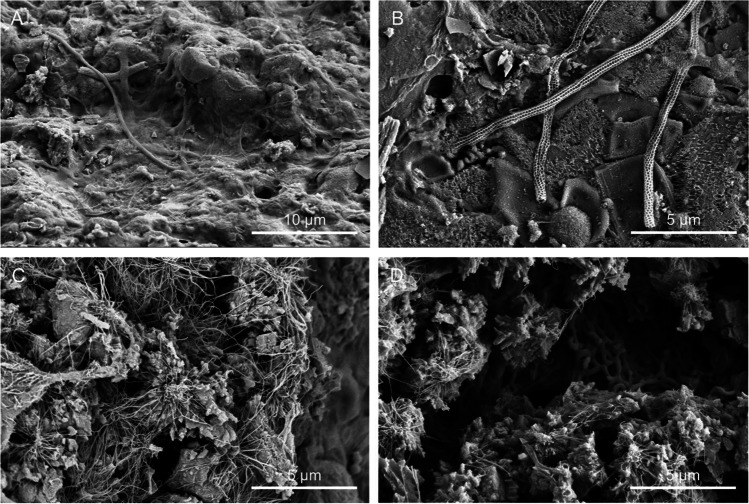
Field emission scanning electron microscopy images of GL samples. Representative field emission scanning electron microscopy images of the studied samples, depicting **A**, **B** reticulated filaments embedded in a matrix of extracellular polymeric substances (GL_1) and **C**, **D** Actinobacteria-like filamentous forms (GL_3)

Microbial cells and structures were imaged by high-resolution FESEM, as an effective and fast method for microbial life detection in complex samples. FESEM images showed abundant microbial cells with different morphotypes in all the samples (Figs. 2, 3, 4, and 5). The most common forms were rod-shaped and coccoidal cells with surface appendages, resembling the actinobacterial cells reported by [3] in lava tubes from USA, Canada, Portugal, and Spain. Samples from “Grotta del Santo,” GS_1A (Fig. 2A, B, C), GS_2A (Fig. 2D), GS_2B (Fig. 2E, F), GC1_1A (Fig. 3A, B), and from “Grotta di Monte Corruccio,” GMC_5 (Fig. 4G, H) showed to be the most abundant in actinobacteria-like cells, as revealed by FESEM.

FESEM observations showed an abundant presence of actinobacteria-like cells for the four samples collected in “Grotta del Santo,” mainly comprising spores with a hairy surface (Fig. 2A–C) or chains of spores with spiny surfaces (Fig. 2D–F). Actinobacteria have been frequently reported in karstic and volcanic caves worldwide (e.g., Riquelme et al. [3]; Porca et al. [41]; Axenov-Gribanov et al. [42]). These bacteria grow from the hyphal, which is an important basis for classification and comparable to filamentous fungi [43]. Other filamentous bacterial structures with smooth surfaces were also found, particularly in sample GS_4, in close association with the mineral substrate (Fig. 2G, H).

Sample collected in “Grotta Catanese I” (GC1_1A) revealed the presence of several actinobacteria-like coccoid (1-µm diameter) and rod-shaped cells (Fig. 3A) with spiny ornamentation, as well as filaments with hairy ornamentation, intermingled with coccoid cells (Fig. 3B). In contrast, sample GC1_2 from the same cave showed a prevalence of diatoms, specifically *Orthoseira roeseana* (Fig. 3C) and *Humidophila* (Fig. 3D), which is related to its location near the cave entrance. It is well documented that at the entrance of caves and on artificially illuminated cave walls, phototrophic organisms mainly cyanobacteria, green algae, and diatoms develop with increasing moisture [44]. In this study, samples of whitish, beige, grey, or yellow bacterial mats that often form on the middle or dark zones of caves were selected and collected, tentatively avoiding phototrophic-based biofilms located at the cave entrances.

Samples collected in “Grotta di Monte Corruccio” showed greater variety in microbial structures and cell morphologies (Fig. 4). In the yellow mat sample (GMC_1), a biofilm of rod-shaped cells, embedded in a matrix of extracellular polymeric substances (EPS) was clearly observed by FESEM (Fig. 4A). Biofilms are multicellular microbial populations that typically adhered to solid surfaces due to the release of EPS, which self-encapsulate the cells and provide structure to biofilms [45]. They comprise a survival strategy of microbial cells to thrive in these hostile environments. Actinobacteria-like spores with hairy ornamentation and spiny surfaces were also observed in all samples collected in this cave (Fig. 4B–H). Sample GMC_4 revealed the presence of microbial imprints, suggesting that the cell-like shapes occur within internal laminae (Fig. 4E), as well as the presence of some clusters of Actinobacteria-like cells and rod-shaped bacteria impregnated in EPS (Fig. 4F). A tangled mass of actinobacteria-like hyphae or archaeal-like cells with hami that protrude from their cell surfaces was observed by Perras et al. [46].

Observations conducted on sample GMC_5 revealed the presence of Ca-rich spheroids closely associated with filamentous cells (Fig. 4G, H), resembling the CaCO_3_ microspheres found in Kipuka Kanohina lava cave in Hawaii, USA [3]. Studies on cave microbial communities revealed that actinobacteria may induce biomineralization processes, promoting nucleation sites or changes in pH [3, 47]. Precipitation of carbonates can be induced by bacteria via urea hydrolysis [48] or through the uptake of carbon dioxide promoting changes in pH and followed by mineral precipitation and CaCO_3_ formation [47]. Numerous biogenic minerals have been reported also in lava caves. Calcite and Mg–silicate minerals were found associated with actinobacterial morphologies on coralloid type speleothems from Chile [7]. Calcium carbonate spheres closely related to dense networks of hairy filaments were observed within the colored microbial mats from Azorean, Canarian, and Hawaiian [3]. Silica microspheres embedded in EPS matrix were observed in Galapagos Islands [16].

Interestingly, sample from “Grotta Lunga” (GL_1) showed the presence of reticulated filaments embedded in a matrix of EPS (Fig. 5A, B). The presence of reticulated filaments has been frequently reported in caves worldwide, in both lava and limestone caves [49–52] but their nature still results enigmatic for microbiologists [54]. FESEM observations of the sample GL_3 revealed a variety of actinobacteria-like filamentous forms spread all over the sample (Fig. 5C, D).

### Richness and Diversity of Cave Microbial Communities

**Fig. 6:**
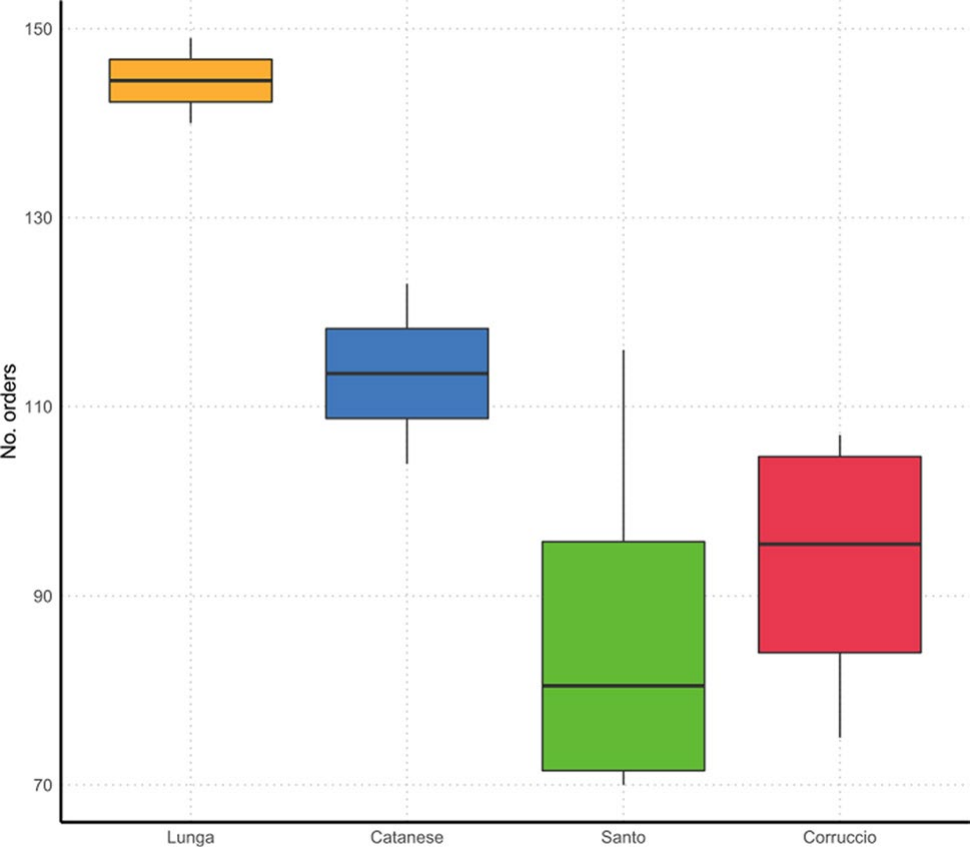
Number of orders of bacteria observed in the four examined caves

We identified a total of 6310 unique ASVs for the 12 samples, with the samples collected in “Grotta Lunga” being richer than the others (GL_3 = 1190 ASVs; GL_1 = 1057 ASVs). These samples also showed the highest values in terms of alpha diversity indices, i.e., Shannon index [54] and Pielou’s evenness [55] (Table 1), calculated on the ASVs (Fig. 6).

**Table 1:** Statistics. Diversity indexes

Cave name	Touristic use	Samples	Raw reads	Trimmed reads	Observed ASVs	Shannon	Pielou’s
Grotta Lunga	High	GL_1	113,047	51,502	1057	8.117803	0.8121241
		GL_3	107,045	48,489	1190	8.762284	0.8618003
Grotta del Santo	High	GS_1	132,755	61,396	621	5.546317	0.6012919
		GS_2A	105,144	21,755	410	5.578653	0.6440540
		GS_2B	128,250	56,174	286	2.527644	0.3113268
		GS_4	132,437	58,838	678	7.348656	0.7855029
Grotta Catanese I	Low	GC1_1A	105,899	43,269	677	5.077743	0.5432766
		GC1_2	124,509	52,204	743	4.797957	0.5055883
Grotta di Monte Corruccio	Low	GMC_1	125,910	55,677	639	4.144329	0.4468860
		GMC_2	108,121	50,496	569	6.867270	0.750339
		GMC_4	141,225	66,672	384	5.293697	0.6199319
		GMC_5	131,544	56,512	405	3.209504	0.3722437

**Fig. 7:**
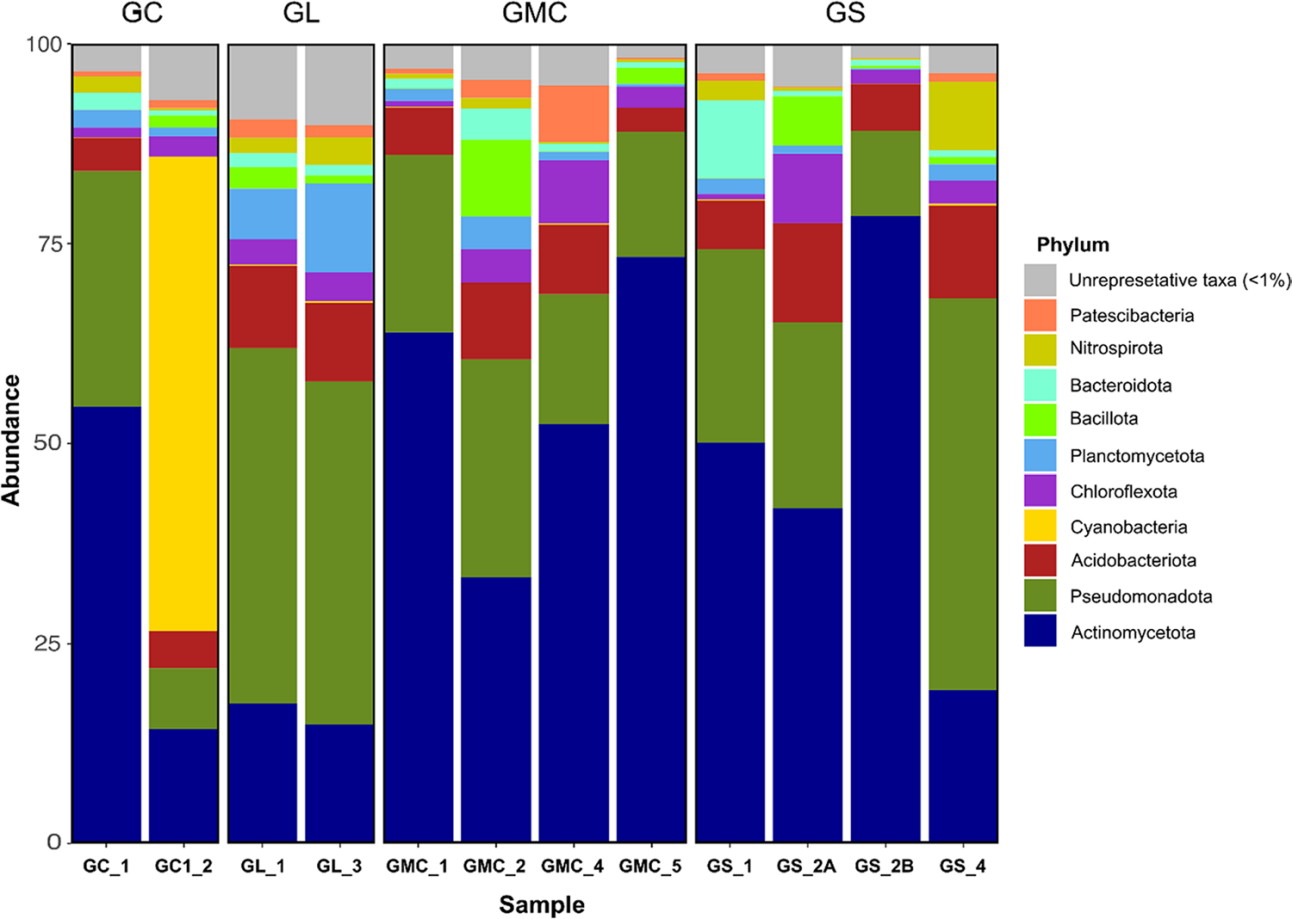
Distribution patterns of bacterial phyla in the samples (99% ASV cutoff) of “Grotta Catanese I” (GC), “Grotta Lunga” (GL), “Grotta di Monte Corruccio” (GMC), and “Grotta del Santo” (GS)

When considering the taxonomic richness measured at the order level, the “Grotta Lunga” resulted the richest cave with 174 orders, followed by “Grotta Catanese I” with 150 orders, and then by “Grotta del Santo” and “Grotta di Monte Corruccio” caves with 148 and 147 orders, respectively (Fig. 7). The result of the GLM showed a significant effect of the cave identity on the taxonomic richness measured at the order level (Chi = 46.4; *P* < 0.001). By comparing the differences among caves with the Tukey’s post hoc test, we could highlight that most of this effect is due to the significant difference between the “Grotta Lunga” and the others, with the former being significantly richer than the others (Lunga – Catanese: *z* = 2.72, *P* = 0.034; Lunga – Santo: *z* = 6.41, *P* < 0.001; Lunga – Corruccio: *z* = 5.59, *P* < 0.001). Regarding the other pairwise comparisons, we could record a significant difference between “Grotta Catanese I” and “Grotta del Santo” (*z* = 3.15, *P* = 0.009), while no other significant differences were identified (|*z*|< 2.34; *P* > 0.05).

Due to the heterogeneity of the examined caves, the small number of samples and considering the pioneer nature of this study, specific patterns are hard to discern, as well as possible relations between the observed parameters of richness and abundance and any environmental factor characterizing the caves we studied. In general terms, major values of richness were observed in “Grotta Lunga” being characterized by low elevation (850 m) and high temperature (18.7 °C). On the contrary, lower values of richness were observed in “Grotta di Monte Corruccio,” which is located at 1365 m, with a temperature of 13.9 °C. Tentatively, differences among caves in terms of richness could also be explained by the different environmental conditions and biogeochemical processes operating in caves [56] as well as a possible response of the microbial communities to human disturbance [57]. Accordingly, richness was higher in “Grotta Lunga” and “Grotta Catanese I” experiencing low tourist use. Similar results were obtained by Ikner et al. [58] reporting lower diversity in caves highly exploited for touristic purposes.

### Microbial Communities

Independent libraries of DNA sequences from each sample were built targeting the V3-V4 hypervariable regions of the 16S rRNA gene, with the objective of detecting the total bacteria present in these samples.

**Fig. 8:**
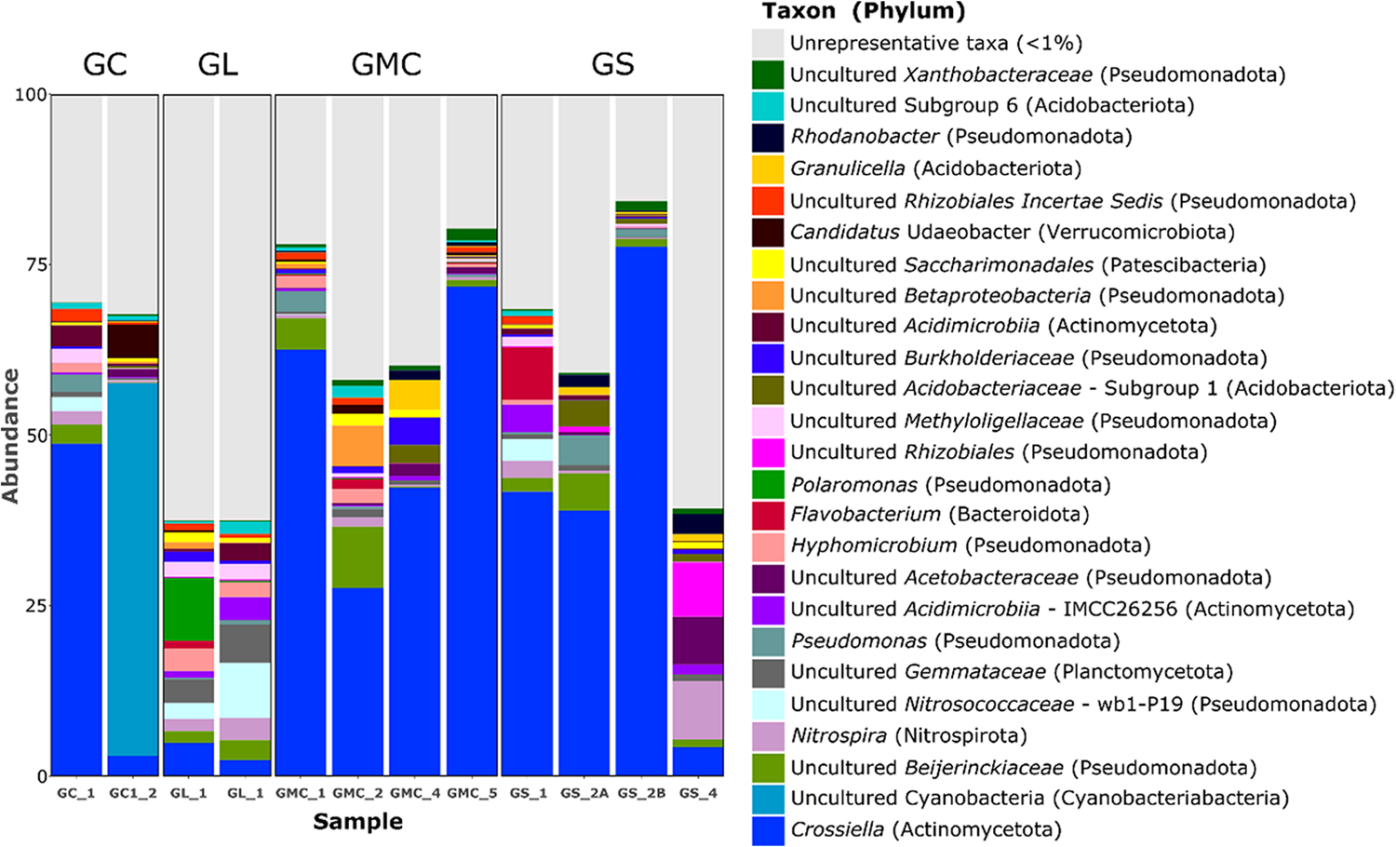
Taxonomic compositions of the 12 samples based on the 16S rRNA gene markers (99% ASV cutoff). Identifications reached the genus level with the exception of ASVs identified as uncultured and candidatus bacteria. In brackets (), the affiliation to phylum was included

The relative abundances of the dominant phyla in each cave sample are shown in Fig. 8. Most of the identified bacteria belonged to *Actinomycetota*, *Pseudomonadota*, *Acidobacteriota*, *Chloroflexota*, and *Cyanobacteria*, followed by other phyla with lower representativeness. Taxonomic identification resulted in a prominent importance of the phylum *Actinomycetota* in almost all samples, ranging from 78.4% in GS_2B to 14.3% in GC1_2, corroborating the observations by FESEM.

The presence of *Actinomycetota* has been widely found in caves where are actively involved in the biomineralization processes (e.g., Riquelme et al. [3]; Barton et al. [59]). Moreover, members of the phylum *Actinomycetota* represent a promising source of bioactive metabolites for drug development [60, 61].

The phylum *Pseudomonadota* was also present in each cave, being the most representative group in “Grotta del Santo” and “Grotta Lunga” (GS_4, GL_1, and GL_3 with 48.97%, 44.43%, and 42.88%, respectively). The *Pseudomonadota* are well represented in lava caves across the world [62], including New Mexico, Hawaii, Azores, and Galapagos Islands [9, 11, 15].

A similar trend was observed by Gonzalez-Pimentel et al. [8] and Miller et al. [15], where the phyla *Actinomycetota* and *Pseudomonadota* were among the most representative groups in lava tubes from La Palma (Canary Islands) and Galápagos (Equador), respectively.

The phylum *Acidobacteriota* was equally distributed in all caves, ranging from 3.02% in “Grotta di Monte Corruccio” (sample GMC_5) and 12.39% in “Grotta del Santo” (sample GS_2A). Several studies have detected the presence of acidobacterial 16S rRNA gene sequences in caves (e.g., Holmes et al. [63]; Hutchens et al. [64]; Chelius and Moore [65]; Engel et al. [66]). However, their role is still poorly understood [67].

The phylum *Cyanobacteria* was abundant in Grotta Catanese I but poorly represented in the other caves. The presence of this taxon in sample GC1_2 is due to its proximity to the cave entrance, which receives sunlight allowing the development of photosynthetic-based biofilms on the cave wall. Accordingly, *Cyanobacteria* are mainly favored by the presence of light and thereby generally frequent near the cave entrance [68, 69], although the presence of artificial light can promote their growth in the innermost cave zones (e.g., Piano et al. [70]), which is not the case. Phyla with less representativeness, but with at least 5% of abundance, were *Chloroflexota*, *Planctomycetota*, *Bacillota*, *Nitrospirota*, *Bacteroidota*, *Patescibacteria*, and *Verrucomicrobiota*. They are also common at lower rates in other lava tubes [3, 6, 9]. Other phyla with relative abundances < 1% were also retrieved.

The bacterial communities identified at the class level showed differences between caves and sectors. Most bacteria belonged to the following class: *Actinomycetes*, *Gammaproteobacteria*, *Alphaproteobacteria*, *Oxyphotobacteria*, followed by the less abundant *Bacilli*, *Bacteroidia*, and *Nitrospira* (Fig. 9).

The relative abundance of the different classes varied in each sample, suggesting differences in the local environment and element composition of the volcanic substrate [71]. *Actinomycetes* was the most abundant class in “Grotta del Santo” (GS_2B: 78.21%) and “Grotta di Monte Corruccio” (GMC_5: 72.76%; GMC_1: 62.94%) sample, while *Gammaproteobacteria* was predominant in “Grotta Lunga” (GL_1: 28.60%) and “Grotta del Santo” (GS_4: 25.99%). *Oxyphotobacteria* were instead the most abundant class in “Grotta Catanese I” (GC1_2). The class *Alphaproteobacteria* was also well represented in all samples, ranging from 22.37% in “Grotta del Santo” GS_4 and 4.66% in “Grotta Catanese I” (GC1_2). The class *Actinomycetes* is common in caves and its studies in different locations have revealed the presence of several novels and rare taxon [3, 8, 57]. Investigations on this taxon have highlighted its biotechnological relevance [72–74] as well as their importance as potential pathogens (e.g., Jurado et al. [75]; Gonzalez-Pimentel et al. [8]; Buresova et al. [76]).

The class *Gammaproteobacteria* is among the most dominant group in habitats with either natural or anthropogenic organic inputs [77]. Accordingly, it seems to represent a good bioindicator to detect the presence of contaminants in soil [78–80] or caves [81]. This taxon was abundant in samples GL_1 (28.69%), GL_3 (24.65%) from “Grotta Lunga” and GS_4 (25.99) from “Grotta del Santo,” which represent “high tourist use” caves. The presence of pathogens in caves has rarely been discussed and evidence of contamination still results scarce (but see Luong et al. [82]). Among pathogens frequently detected in caves, considered indicators of human impact, there are *Bacillus* spp., *Escherichia coli*, and *Staphylococcus aureus* [81, 83, 84].

The bacterial communities identified at the genus level showed differences between caves and sectors. The genus *Crossiella* is well present in all samples, being the most abundant group in 8 out of 12 analyzed samples (Fig. 8). The taxon was abundant in sample GS_2B from “Grotta del Santo,” and GMC_1 and GMC_5 from “Grotta di Monte Corruccio” with a relative abundance respectively of 77.6%, 71.8%, and 62.54%. The genus is common in lava tube caves of Hawaii and Azores [3, 11, 85] and also in limestone caves [86]. Gonzalez-Pimentel [17] observed a high abundance of *Crossiella* in moonmilk from La Palma lava caves. Recent studies have hypothesized the influence of *Crossiella* on the nitrogen cycle and its possible contribution to calcium carbonate precipitation in caves [87, 88].

Some important groups after *Crossiella* were the genus *Pseudomonas*, *Bacillus*, *Chujaibacter*, and *Sphingomonas*. *Nitrospira* and the family groups *Beijerinckiaceae* and *Nitrosococcaceae* are respectively described as nitrite-oxidizing, nitrogen fixation, and ammonia-oxidizing bacteria, relatively common in caves [6, 9, 11, 89]. *Chujaibacter* is associated with heavy metal metabolism [90]. The information provided by the identification of these groups of bacteria could respond to the presence of the influence of vegetation and anthropic pressure on the studied caves.

The presence of *Bacillus*, *Pseudomonas*, and *Sphingomonas* in caves is often associated with the presence of high human impact (e.g., Lavoie and Northup [83]; Ikner et al. [58]; Bastian et al. [81]) and recent studies have showed their antimicrobial activity against pathogenic bacteria, as well as a potential source of new microorganism [91, 92]. Other genera identified were as follows: *Alkanibacter*, *Flavobacterium*, *Steroidobacter*, *Sporosarcina*, wb1-P19, *Polaromonas*, this last in clone libraries and culture collections from polar and high-elevation environments [93].

## Conclusions

This study provides the first insight into the taxonomic groups constituting the microbial communities of Mount Etna lava tubes. Although the limited number of samples did not allow us to properly correlate these data with any environmental variables, we could detect the higher richness in “Grotta Lunga,” which is located at a lower elevation (850 m a.s.l.), whereas we observed a general lower richness at higher elevation (1365 m a.s.l.) in “Grotta di Monte Corruccio.” Accordingly, differences among caves in terms of richness could be explained by the different environmental conditions and biogeochemical processes operating in caves [55]. Further investigations would be therefore desirable to shed light on which factors drive the richness of microbial communities on Mount Etna lava caves.

Our results revealed the abundant presence of phylotypes previously detected in other lava tubes worldwide. The presence of a large number of unclassified bacteria in the 12 sampling sites suggests that these lava tubes have a great potential for the isolation of novel species. However, the land use overlying the cave, as well as the uncontrolled presence of visitors into these caves, may pose major threats to these ecosystems, especially those located at lower altitudes beyond the strictly protected areas of the volcano, generally exposed to a higher tourist frequentation. Further research is needed to ascertain the possible presence of novel bacteria in caves from the Etna lava caves, as well as the presence of contaminants and their potential risk associated with human health. Data of pathogens should be therefore considered not only for the conservation of these unique geosites, but also for the risk associated with human health.

## Supplementary Information

Below is the link to the electronic supplementary material.

Supplementary file1 (DOCX 1180 KB)

## Data Availability

The data that support the findings of this study are available from the corresponding author upon reasonable request.
