# Supplementary material for: First Insights into the Bacterial Diversity of Mount Etna Volcanic Caves
Source: Microb Ecol. 2023 Feb 8;86(3):1632–45. doi: 10.1007/s00248-023-02181-2 (PMC10497698; doi:10.1007/s00248-023-02181-2)
Supplement: Supplementary file 1 — Supplementary file1 (DOCX 1180 KB) [file 248_2023_2181_MOESM1_ESM.docx]

**Table S1**. List of the 4 caves investigated. The main morphological and environmental variables are reported in the columns. The geological setting is derived from Branca et al., 2011. Results of bulk rock analyses for major (wt.%) and trace (ppm) elements are derived from Matteoni, 2013 (“Grotta Lunga”) and Lanzafame & Ferlito, 2014 (“Grotta del Santo”).

| **Lava cave** | **Cadastrial number** | **Coordinates** | **Altitude (m a.s.l.)** | **Total cave length (m)** | **Altitude (m a.s.l.)** | **Temperature (°C) / Relative humidity** | **Age (years)** | **Geological setting** | **Bulk chemistry data** |
| --- | --- | --- | --- | --- | --- | --- | --- | --- | --- |
| “Grotta del Santo”  (or “San Nicola”) | SICT1032 | 37.709182, 14.875877 | 1030 | 924 | 1030 | 14ºC / >70% | 15ka - 3960±60 a | Lava flows, scoria cones, spatter ramparts and pyroclastic fall deposits related to flank and summit eruptions occurred between post-Ellittico caldera (15 ka) and FS lithohorizon (radiocarbon age 3960±60 a). | Major elements (wt.%): SiO2 = 52.22, TiO2 = 1.53, Al2O3 = 16.78, FeO = 8.16, Fe2O3=1.22,  MnO = 0.16, MgO = 2.48, CaO = 9.07, Na2O = 3.43, K2O = 1.96, P2O5 = 0.77, LOI=1.36. Trace elements (ppm): Rb=38, Ba=655, Th=5, Nb=54,  La=57, Ce=122, Sr=947, Zr=200, Y=25, V=121, Pb=26, Cr=19, Ni=15,  Zn=46, Co=40. |
| “Grotta Catanese I” | SICT1037 | 37.648333, 14.939167 | 905 | 145 | 905 | 15.5ºC / >70% | 500 BC | Lava flows, scoria cones, spatter ramparts and pyroclastic fall deposits related to flank and summit eruptions occurred between post-FS lithohorizon (radiocarbon age 3960±60 a and Il Piano caldera (122 BC plinian eruption). | - |
| “Grotta di Monte Corruccio” | SICT1056 | 37.810389, 15.075167 | 1365 | 81 | 1365 | 13.9ºC / >70% | 15ka - 3960±60 a | Lava flows, scoria cones, spatter ramparts and pyroclastic fall deposits related to flank and summit eruptions occurred between post-Ellittico caldera (15 ka) and FS lithohorizon (radiocarbon age 3960±60 a | - |
| “Grotta Lunga” | SICT1029 | 37.629734, 15.035672 | 850 | 53 | 850 | 18.7ºC / >80% | 300±100 AD | Lava flows characterised by aa morphologies and, rarely, pahoehoe. Lava composition ranges from basalt to mugeariteaphyric to highly porphyritic texture, with phenocrysts of pl, px, ol, in variable quantity and size.  Volcanics belonging to this formation are displayed in 3 time intervals: (1) post- 122 BC plinian eruption - 1669 AD eruption; (2) post 1669 AD eruption - pre 1971 AD eruption; (3) 1971 AD eruption – present (May 2007 eruption). | Major elements (wt.%): SiO2 = 48.82, TiO2 = 1.59, Al2O3 = 17.84, Fe2O3=10.26, MnO = 0.15, MgO = 4.75, CaO = 9.55, Na2O = 3.9, K2O = 1.75, P2O5 = 0.59,  LOI=-0.05.  Trace elements (ppm): Rb=35.8, Ba=797, Th=5, Nb=55.4, La=71, Ce=140, Sr=1141, Zr=197, Y=26.8, V=265, Pb=26, Cr=23, Ni=20, Zn=46, Co=33. |

**Table S2**. Sampling site description for each studied cave.

| **Lava cave** | **Sample codes** | **Sampling site photo** | **Stereomicroscope photo** | **Distance from cave entrance (m)** | **Sample description and color** | **Remarks** |
| --- | --- | --- | --- | --- | --- | --- |
| “Grotta del Santo” | GS_1A | 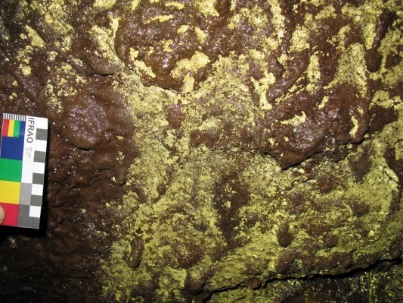 | 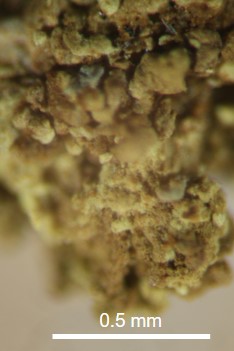 | 50 | yellow | Dense mats colonies spread in the innermost cave sectors |
|  | GS_2A | 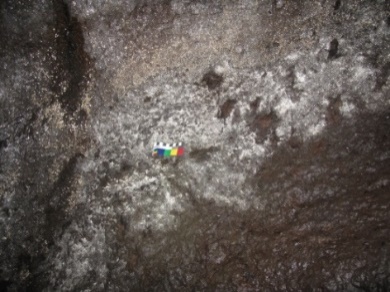 | 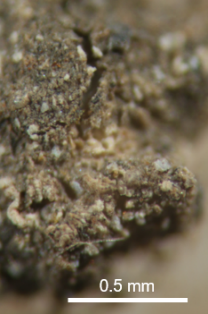 | 60 | white - beige | Dense mats colonies spread in the innermost cave sectors |
|  | GS_2B | 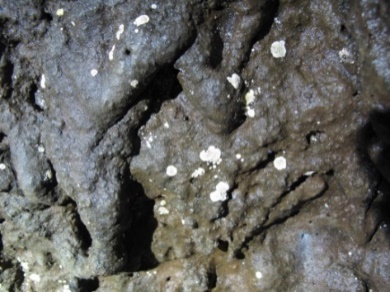 | 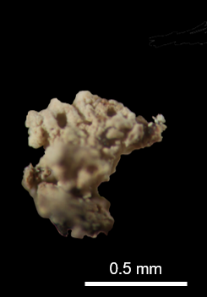 | 55 | beige | Wide scattered colonies spread throughout the cave |
|  | GS_4 | 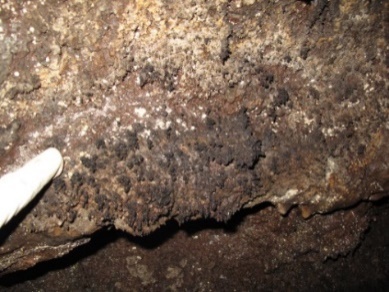 | 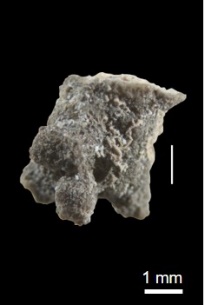 | 20 | black coralloids | Spread in the innermost cave sectors |
| “Grotta Catanese I” | GC1_1A | 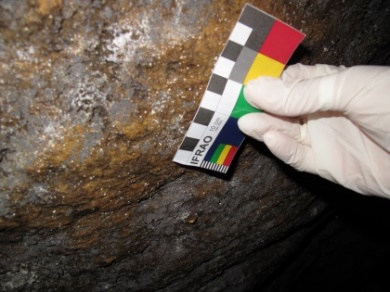 | 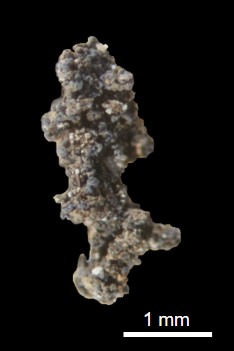 | 50 | grey | Dense mats colonies spread in the central cave sectors |
|  | GC1_2 | 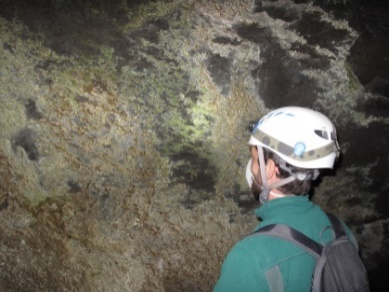 | 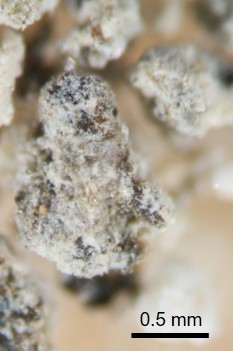 | 20 | green-reddish | Dense mats colonies spread in the outermost cave sectors |
| “Grotta di Monte Corruccio” | GMC_1 | 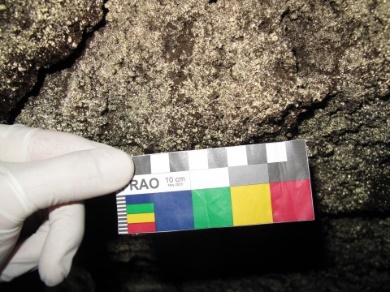 | 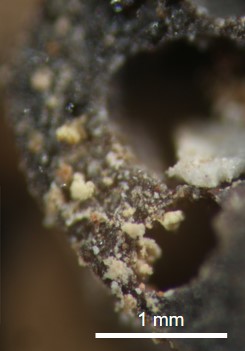 | 22 | yellow | Dense mats colonies spread in the innermost cave sectors |
|  | GMC_2 | 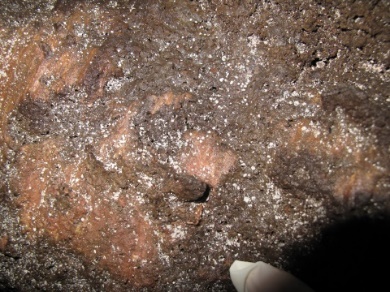 | 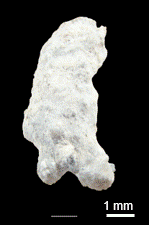 | 20 | white | Small scattered colonies spread throughout the cave |
|  | GMC_4 | 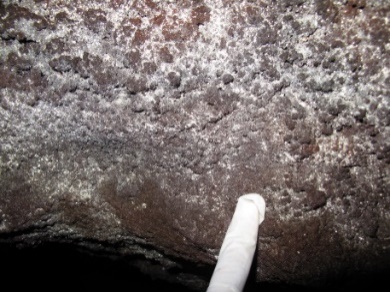 | 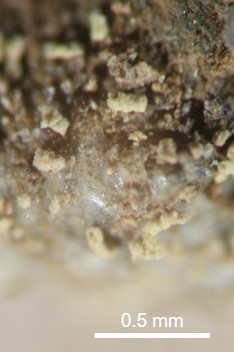 | 20 | white | Dense mats colonies spread in the innermost cave sectors |
|  | GMC_5 | 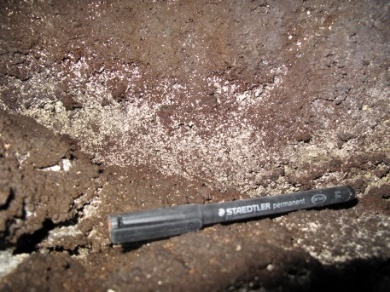 | 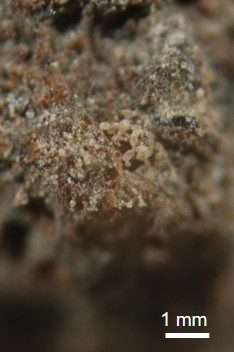 | 20 | beige | Dense mats colonies spread in the innermost cave sectors |
| “Grotta Lunga” | GL_1 | 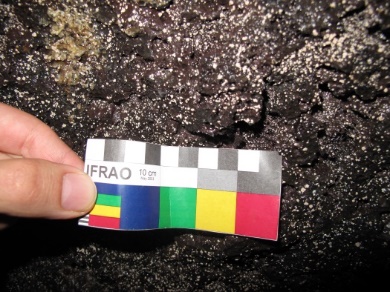 | 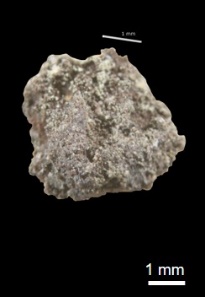 | 40 | white | Dense mats colonies spread throughout the cave |
|  | GL_3 | 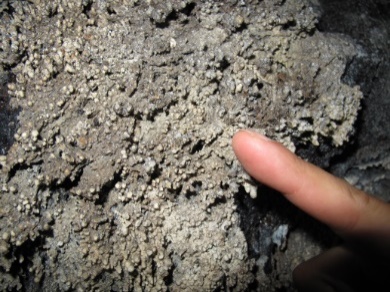 | 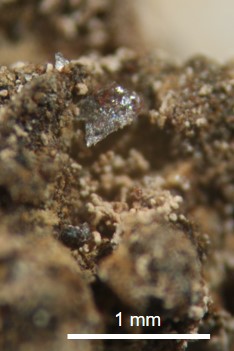 | 20 | light grey -white | Small scattered colonies spread throughout the cave |
